# Salivary Gland Pleomorphic Adenomas Presenting With Extremely Varied Clinical Courses. A Single Institution Case-Control Study^†^

**DOI:** 10.3389/fonc.2020.600707

**Published:** 2021-01-08

**Authors:** Krzysztof Piwowarczyk, Ewelina Bartkowiak, Paweł Kosikowski, Jadzia Tin-Tsen Chou, Małgorzata Wierzbicka

**Affiliations:** ^1^ Department of Otolaryngology and Laryngological Oncology, Poznan University of Medical Sciences, Poznan, Poland; ^2^ Department of Clinical Pathology, Poznan University of Medical Sciences, Poznan, Poland

**Keywords:** mixed tumor, parotid gland tumor, recurrence, surgery, progression, facial nerve

## Abstract

**Objective:**

Pleomorphic adenomas (PAs) with divergent clinical behavior, differing from the vast majority of PAs, were distinguished. “Fast” PAs are characterized by an unexpectedly short medical history and relatively rapid growth. The reference group consisted of “slow” PAs with very stable biology and long-term progression. We divide the PA group as a whole into three subsets: “fast,” “normal,” and “slow” tumors. Our goal is a multifactorial analysis of the “fast” and “slow” PA subgroups.

**Methods:**

Consecutive surgeries in a tertiary referral center, the Department of Otolaryngology and Laryngological Surgery, Poznan University of Medical Sciences, Poland, were carried out between 2002 and 2011. Out of 1,154 parotid tumors, 636 (55.1%) were PAs. The data were collected prospectively in collaboration with the Polish National Registry of Benign Salivary Gland Tumors. The main outcome measure was the recurrence rate in “fast” and “slow” PA subgroups. All surgical qualifications and surgeries were performed by two experienced surgeons.

**Results:**

Slow PAs, compared to fast PAs, presented in older patients (53.25 ± 15.29 versus 47.92 ± 13.44 years). Multifactor logistic regression analysis with recurrence (yes/no) as the outcome variable, fast/slow as the predictor variable and age, gender, margin, FN status as covariates showed that fast PAs were significantly predicting recurrence vs. slow PAs (p = 0.035). Fast PAs were increasing the risk of PAs 10-fold vs. slow PAs, exp β = 10.20, CI_95_ [1.66; 197.87]. The variables impacting relapse were recent accelerated growth of the tumor OR = 3.35 (SE = 0.56), p = 0.030, positive margins OR = 7.18 (SE = 0.57), p < 0.001, incomplete or bare capsule OR = 9.91 (SE = 0.53), p = 0.001 and location III OR = 3.12 (SE = 0.53), p = 0.033. In the multivariate model only positive margin was selected as the best predictor of relapse, OR = 5.01 (SE = 0.60), p = 0.007.

**Conclusions:**

The simple clinical aspect of slow or fast PA progression is of great practical importance and can constitute a surrogate of the final histopathological information that is derived from the surgical specimen. The slow or fast nature of the PA to some extent indicates prognostic features such as recurrence risk. This finding requires correlation with histological and molecular features in further stages of research.

## Introduction

Pleomorphic adenomas (PA) are the most common parotid tumors and their trend of incidence is increasing (1, 2). These tumors are slow-growing and can remain asymptomatic and unrecognized, or unobtrusive enough that the patient decides not to undergo treatment. Though they may reach significant size over a period of years, some of them present misleadingly short histories constituting rather rapid development (3, 4).

It is important to accurately establish the histology of all benign salivary tumors in order to predict their clinical behavior, and this is particularly true in the case of PA due to its histological variants, different tumor entities, and the possibility of treatment failure (5, 6). The post-operative incidence of PA recurrence is significant and varies largely because of differences in surgical technique (1, 7, 8), as well as other factors including multi-nodularity and pseudopodia, tumor diameter, the age of the patient, and cellular and molecular changes (9–12). The risk of malignant transformation to carcinoma ex pleomorphic adenoma (Ca ex PA) occurs in only 1.8–6.2% of cases (13, 14), with a prevalence rate of 5.6 cases per 100,000 malignant tumors and an incidence rate of 0.17 tumors per million persons (15).

The histological diagnosis of the majority of PAs is straightforward. The tumor is usually well-circumscribed, encapsulated with a bosselated outer surface, and often presents with tongue-like protrusions or sometimes satellite nodules. Morphological patterns vary, with typically the following three components present: (1) epithelial and (2) myoepithelial cells, with (3) areas of mesenchymal differentiation. There are varying proportions of tubules, duct-like structures, and mesenchymal tissues (16) and different histological patterns of myoepithelial cells, which may appear as plasmacytoid, spindle, epithelioid, clear, or stellate (16, 17). Metaplastic changes and the foci of squamous cells are an integral feature of PAs, however extensive squamous metaplasia is uncommon and can easily be misinterpreted as squamous cell carcinoma (18).

Morphologic and genetic studies on PAs are scarce and there are still gaps in the knowledge concerning variations in clinical behavior and adverse outcomes (19). Furthermore, no pathological features of the tumor are available prior to surgery. We know only the tumor’s dimensions and the duration and speed of its growth. Our experience with 1,154 benign salivary gland tumors over a 10-year period has prompted us to distinguish a small group of PA tumors with clinical behavior that differs from the vast majority of PA. Progression, recurrence, and malignant transformation are well-known PA behavior, but the unusually fast growth of this benign tumor has always surprised clinicians. The impact of this phenomenon on the treatment failure rate is unknown. Our goal is a multifactorial analysis of fast versus slow PA tumors, with the main end result being recurrence and the main outcome measure being the correlation of this failure with the clinical nature of the tumor (slow/fast), tumor size, tumor volume, and additional factors such as age, gender, margins, and facial nerve (FN) status.

## Materials and Methods

In total, 1,154 benign parotid tumors were consecutively operated on in a tertiary referral center, the Department of Otolaryngology and Laryngological Surgery, Poznan University of Medical Sciences, Poland, between 2002 and 2011. Of these, 636 (55.1%) were PA. The data were initially collected prospectively from a local database and, from 2015 onwards, from the Polish National Registry of Benign Salivary Gland Tumors. There were 224 (35.2%) men, 412 (64.8%) women, with ages ranging from 13 to 86 years, mean 47.93 ± 14.93 years and median 48 years. All patients were operated on by two experienced surgeons (MW, TK).

This study was conducted in accordance with a protocol approved by the Bioethics Committee of Poznan University of Medical Sciences (Resolution No. 781/16), and written consent was obtained from each patient.

### Clinically “Fast” and “Slow” Tumors

The PA group was divided into three subsets: “fast,” “normal/stable,” and “slow” tumors, based on several clinical and radiological features. Three different criteria were used to categorize tumors. Objective criteria were history-based growth time and growth rate, determined by tumor increment in percent by volume, as per the patient’s description. The subjective criteria were the radiological features assessed by the doctor in one of the imaging modalities, predominantly ultrasonography. “Slow” tumors had over 10 years’ history and exhibited slow growth (<5% of tumor size over the last 10 years). “Stable” tumors constitute the vast majority of PA and are characterized by anamnesis >=3 years, stable size of the tumor or its slow growth (<5% of tumor size over the last 6 months); a well-visualized tumor capsule in the radiological investigation, and tumor homogeneity. The “fast” tumors are characterized by an unexpectedly short medical history and relatively rapid growth. The criteria were as follows: anamnesis <3 years; >5% growth of the tumor size within six months; and multi-polycyclic outline, heterogenic echostucture and loss of capsule echogenicity in radiological investigation. To accurately and unequivocally categorize a tumor as “fast,” all the criteria had to be obtained.

### Variables Collected for PAs

The variables age, sex, place of residence, time between first symptoms and surgery, tumor location, margins, FN status after surgery, and recurrence were collected.

Tumor location was presented according to the European Salivary Gland Society’s (ESGS) classification of salivary gland surgeries (2, 20). The ESGS operative report includes the level removed, designated by the Roman numerals I to V in ascending order, and non-glandular structures removed, each identified through the use of specified acronyms.

Surgical approach. The classification of salivary gland surgeries was presented according to the ESGS (2, 20) and distinguishes two types of surgery: extracapsular dissection and parotidectomy. The ESGS operative report includes the glandular parenchyma level removed, designated by Roman numerals I to V. Extracapsular dissection, partial superficial parotidectomy, superficial parotidectomy, and total parotidectomy were noted.

Margins. In benign salivary gland tumors, there is no concept of positive or negative margins as there would be in malignant cancers. Positive margins were categorized by the following adverse findings: capsular rupture and intra-operative tumor spillage, the presence of incomplete or bare capsule or absence of encapsulation in the pathology specimen, and satellite nodules as distinct tumor nodules.

FN status. Function of the facial nerve using the House-Brackmann scale was recorded at 1 week, 1 month, and 12 months.

Follow up. Routine follow-up is based on ultrasonography performed once a year. In cases with a higher risk of recurrence, ultrasound is performed twice a year, and an additional MRI once a year if needed.

Furthermore, tumor features such as growth rate, capsule visualization in pre-operative imaging, and tumor homogeneity were taken into consideration. The main predictive value was categorization into “fast,” “normal,” and “slow” PA.

The outcome measure was the correlation of recurrence with tumor size, volume, and of recurrence with PA nature (“fast,” “normal,” and “slow”). The main outcome measure was the determination of whether tumor size, tumor nature (slow/fast), or the other variables influenced recurrence more. Subsequent multivariant analysis included additional factors such as age, gender, margins, and FN status.

### Statistical Analysis

Analysis was conducted using R software version 3.5.1. Nominal variables are presented as n (% of group), and continuous variables as mean ± SD or median (Q1;Q3). Normality of distribution was validated using the Shapiro-Wilk test as well as a visual assessment of histograms, skewness, and kurtosis values. Comparison of fast and slow PA groups was conducted with a chi-square test or chi-square test with Yate’s correction for nominal variables and with t-test or Mann-Whitney U test for continuous variables, as appropriate. The mean/median difference (MD) with 95% confidence interval was calculated for continuous variables. To verify the impact of fast/slow PAs on recurrence, a multifactorial logistic regression model was calculated, with age, sex, margins, and FN status as covariates. Model assessment was conducted with the Hosmer–Lemeshow goodness of fit (GOF) test. Additionally, relapse-free survival (RFS) was calculated using Kaplan-Meier survival analysis, including 95% confidence interval. RFS stratified by independent variables (i.e., sex, location, margin, etc.) was compared with log-rank chi-square test. Cox regression model with Breslow method was used to identify parameters impacting relapse. First, univariate models were prepared for each of the independent variables, and based on those models, variables with p < 0.2 in Wald test were included to the final multivariate model. For location variables, due to their inter-dependence, location with the lowest p-value in univariate models was included in the final model. For the margins variable (positive/negative) and the reasons for positive variable: due to inter-dependence of both variables, the final model included the variable (positive/negative) that had a lower p-value in the univariate model. The final multivariate model was created using a stepwise approach. All tests were based on α = 0.05.

## Results

Of the 636 PAs over a 10-year period, there were 84 (13.2%) fast, 73 (11.5%) slow, and 479 (75.3%) normal/stable PAs. The recurrence rate was 8.2% (52/636). All recurrences were ipsilateral. There was no difference in the frequency distribution of individual groups over the years.

There was also a statistically significant relationship between fast/slow PAs and tumor volume (p = 0.033). Smaller tumors (≤ 4 cm^3^) were more frequent with slow PAs (72.6%) vs. fast PAs (52.4%). (**Table 1**).

**Table 1:** Comparison of criteria determining fast and slow PA categorization.

Characteristic	Normal PA	Fast PA	Slow PA	Recurrence (Yes)	MD (95% CI)	p
N	479	84	73	52		
Greatest dimension [cm], mean ± SD	2.42 ± 1.07	2.60 ± 1.06	2.23 ± 0.90	2.15 ± 1.37	0.37 (0.06; 0.67)	0.021
Ratio of the greatest dimension to time*	0.22 ± 0.16	0.34 ± 0.42	0.05 ± 0.02	0.34 ± 0.29	0.29 (0.20; 0.38)	<0.001
Ratio of volume to time*	0.22 (0.08;0.55)	0.40 (0.12;0.84)	0.03 (0.01;0.09)	0.19 (0.12;0.73)	0.37 (0.15; 0.48)	<0.001
Capsule presence, n (%)	293 (61.2)	0 (0.0)	73 (100.0)	7 (13.5)		<0.001
Heterogeneous tumor, n (%)	89 (18.6)	71 (84.5)	0 (0.0)	38 (73.1)		<0.001
Polycyclic outline, n (%)	152 (31.7)	49 (58.3)	9 (12.3)	49 (94.2)		<0.001
Capsule presence + heterogeneous tumor, n (%)	0 (0.0)	0 (0.0)	0 (0.0)	3 (5.8)		>0.999
Capsule presence + polycyclic outline, n (%)	69 (14.4)	0 (0.0)	9 (12.3)	7 (13.5)		0.003
Heterogeneous tumor + polycyclic outline, n (%)	58 (12.1)	45 (53.6)	0 (0.0)	37 (71.2)		<0.001
Capsule presence + heterogeneous tumor + polycyclic outline, n (%)	0 (0.0)	0 (0.0)	0 (0.0)	3 (5.8)		>0.999

MD, mean/median difference between fast/slow groups with 95% confidence interval; p, comparison of fast/slow groups (chi-square test for nominal variables or t-test/Mann-Whitney U test for continuous variables); *, time between first symptoms and surgery, in months.

Next, we analyzed the categories of fast/slow tumors and the correlations with patient epidemiological data and tumor features: tumor location in individual regions of the salivary gland, margins, and condition of the facial nerve after surgery.

The time elapsed between the first symptoms and surgery was significantly different between fast (11.85 ± 6.47 months) and slow (52.03 ± 13.76 months) PA, MD = -40.18 CI_95_ [-43.50; -36.86]; (p < 0.001). There was no significant relationship between slow vs. fast PA and sex or place of residence. Slow PAs presented in older patients (53.25 ± 15.29 years vs. 47.92 ± 13.44 for fast PAs), MD = -5.33 CI_95_ [-9.90; -0.76]; (p = 0.021).

**Figure 1:**
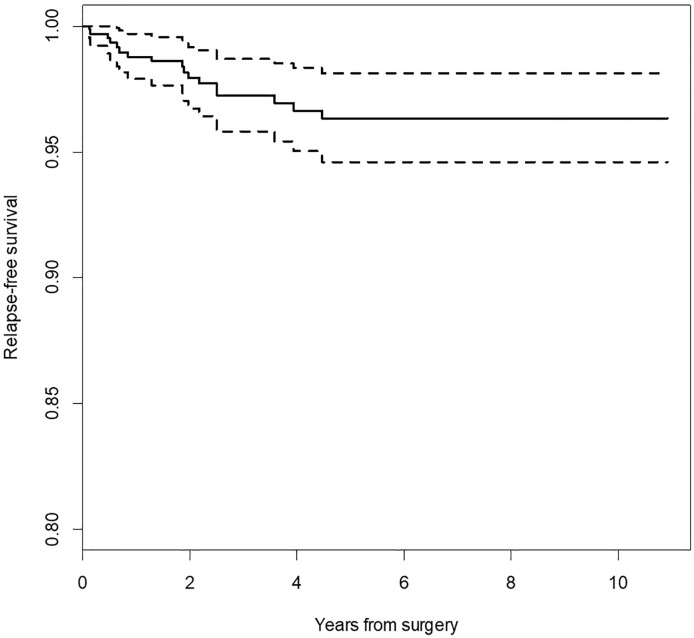
Kaplan-Meier survival curve for relapse-free survival (RFS). Dotted lines indicate 95% confidence interval for survival curve.

Relapse-free survival (RFS) for the whole group was 96.3% CI_95_ [94.6%; 98.1%]. RFS was significantly different in regard to the pace of recent rapid tumor growth (log-rank p = 0.020), positive/negative margins (log-rank p < 0.001), the reason for positive margin (log-rank p < 0.001), location of the tumor in area III (log-rank p = 0.020), and location in area V (log-rank p = 0.020). Log-rank test did not confirm statistically significant differences in RFS for the remaining variables (sex, FN status, location of the tumor in area I, II, IV, I–II, III–IV, parotidectomy type). **Figure 1** demonstrates that the recurrence risk increased during the first 4.2 years after surgery and stabilized after this time.

Localization of the tumor in area I, as designated by the ESGS classification, was significantly more frequent in slow PAs (63.0% vs. 45.2%, p = 0,012) while localization in areas II, III, and IV were more frequent in fast PAs (78.6%% vs. 52.1%, p < 0.001 for location II, 28.6% vs. 4.1%, p < 0.001 for location III, 14.3% vs. 0%, p = 0.002 for location IV). There was no significant relationship between location V and fast/slow PAs. Locations I and II combined, as well as locations I–IV combined were not significantly different when comparing fast vs slow PAs. However, locations III and IV combined were more frequent in fast PAs (33.3% vs. 4.1%, p < 0.001).

FN dysfunction of the marginal-mandibular branch occurred in 35 (7.3%) normal PAs, 18 (21.4%) fast Pas, and 3 (4.1%) slow PAs. Patients of 17 (3.5%) normal PAs, 9 (9.7%) fast Pas, and 2 (2.7%) slow PAs recovered facial function at 1 month; 12 (2.5%) normal PAs, 7 (8.3%) fast Pas, and 1 (1.4%) slow PA recovered facial function at 6 months, and 100% had recovered at 12 months. There were no cases of definitive involvement of FN.

The main outcome measure was the correlation of treatment failure, that is, recurrence with examined variables, with special regard to the fast/slow PA nature. Thus, the key question was, Which of the clinical parameters, age of onset, tumor volume, tumor growth rate, surgical approach, better correlated with a higher risk of recurrence? Based on univariate Cox regression models presented in **Table 2**, variables that were significantly impacting relapse were recent rapid tumor growth, OR = 3.35 (SE = 0.56), p = 0.030, positive margins vs. negative, OR = 7.18 (SE = 0.57), p < 0.001, incomplete or bare capsule vs. other reasons of positive margin, OR = 9.91 (SE = 0.53), p = 0.001 and location III vs. other, OR = 3.12 (SE = 0.53), p = 0.033. In the multivariate model only positive margin was selected as the best predictor of relapse, OR = 5.01 (SE = 0.60), p = 0.007.

**Table 2:** Cox regression model for relapse.

Characteristic	Univariate models			Multivariate model		
	OR	SE	p	OR	SE	p
Age, years	1.002	0.02	0.886			
Sex, male	2.04	0.49	0.142			
Tumor volume [cm^3^]	0.99	0.02	0.844			
Recent rapid tumor growth	3.35	0.56	0.030			
Margins, positive	7.18	0.57	<0.001	5.01	0.60	0.007
Incomplete or bare capsule	9.91	0.53	0.001			
FN status, intact	1.50	1.03	0.694			
Tumor location by ESGS						
I	0.82	0.49	0.684			
II	1.46	0.51	0.457			
III	3.12	0.53	0.033			
IV	2.14	0.75	0.313			
V	7.26	1.03	0.055			
I-II	0.63	0.75	0.542			
III-IV	2.31	0.53	0.116			
I-IV	0.01	0.01	0.997			
Parotidectomy type, ECD = baseline						
Partial superficial	0.87	0.56	0.800			
Superficial	0.44	0.80	0.304			
Total	1.66	0.80	0.528			
ND	0.01	0.01	0.998			

OR, odds ratio; SE, standard error; ND, no data available.

As the two surgeons (MW, TK) performed all surgical qualifications as well as all surgeries, it can be assumed that such a standardization of surgical technique, considered for a given type of surgery, had a limited impact on the incidence of recurrence.

The analysis of fast and slow PA with special regard to recurrence is presented in **Table 3**. The relationship between fast/slow PAs and margins, condition of the FN after surgery, recurrence rate was significant. Positive margins were more frequent in fast PAs (47.9% vs. 17.4% of slow PAs, p < 0.001), and intact FN was also more frequent in fast PAs (21.4% vs. 4.1% of slow PAs, p = 0.001). PAs recurred in 17.9% of fast PAs vs. 1.4% of slow PAs (p = 0.002).

**Table 3:** Analysis of fast and slow pleomorphic adenoma (PA).

Characteristic	Normal PA	Fast PA	Slow PA	Recurrence( Yes)	MD (95% CI)	p
N	479	84	73	52		
Time between first symptoms and surgery, in months (mean ± SD)	13.67 ± 6.21	11.85 ± 6.47	52.03 ± 13.76	11.32 ± 13.16	-40.18 (-43.50;-36.86)	<0.001
Sex, n (%)						
Female	311 (64.9)	52 (61.9)	49 (67.1)	29 (55.8)		0.496
Male	168 (35.1)	32 (38.1)	24 (32.9)	23 (44.2)		
Age, in years (mean ± SD)	47.13 ± 14.99	47.92 ± 13.44	53.25 ± 15.29	48.04 ± 14.14	-5.33 (-9.90;-0.76)	0.021
Place of residence, n (%)						
Rural area	104 (21.7)	14 (16.7)	14 (19.2)	5 (9.6)		0.682
City	375 (78.3)	70 (83.3)	59 (80.8)	47 (90.4)		
Imaging examinations. n (%)						
CT	115 (24.0)	40 (47.6)	29 (39.7)	10 (62.5)		0.268
MRI	44 (9.2)	26 (31.0)	20 (27.4)	37 (231.3)		
US	320 (66.8)	18 (21.4)	24 (32.9)	5 (31.3)		
Tumor location by ESGS, n (%)						
I	290 (60.5)	38 (45.2)	46 (63.0)	31 (59.6)		0.012
II	290 (60.5)	66 (78.6)	38 (52.1)	35 (67.3)		<0.001
III	82 (17.1)	24 (28.6)	3 (4.1)	16 (30.8)		<0.001
IV	30 (6.3)	12 (14.3)	0 (0.0)	7 (13.5)		0.002
V	20 (4.2)	4 (4.8)	1 (1.4)	3 (5.8)		0.434
I-II	435 (90.8)	79 (94.0)	67 (91.8)	49 (94.2)		0.579
III-IV	98 (20.5)	28 (33.3)	3 (4.1)	17 (32.7)		<0.001
I-IV	452 (94.4)	82 (97.6)	67 (91.8)	51 (98.1)		0.097
Parotidectomy type, n (%)						
Partial superficial	163 (34.0)	17 (20.2)	15 (20.5)	14 (26.9)		<0.001
Superficial	100 (20.9)	33 (39.3)	31 (42.5)	11 (21.2)		
Total	31 (6.5)	22 (26.2)	1 (1.4)	12 (23.1)		
ECD	185 (38.6)	10 (11.9)	21 (28.8)	14 (26.9)		
Other	0 (0.0)	2 (2.4)	5 (6.8)	1 (1.9)		
Tumor volume [cm^3^], n (%)						
≤ 4	286 (59.7)	44 (52.4)	53 (72.6)	39 (75.0)		0.033
4-15	148 (30.9)	31 (36.9)	16 (21.9)	9 (17.3)		
≥15	45 (9.4)	9 (10.7)	4 (5.5)	4 (7.7)		
Margins, n (%)						
Positive:	130 (31.7)	35 (47.9)	12 (17.4)	34 (70.8)		<0.001
Negative	280 (68.3)	38 (52.1)	57 (82.6)	14 (29.2)		
FN status, n (%)						
Other	35 (7.3)	18 (21.4)	3 (4.1)	25 (48.1)		0.001
Intact	444 (92.7)	66 (78.6)	70 (95.9)	27 (51.9)		
Recurrence, n (%)						
Yes	36 (7.5)	15 (17.9)	1 (1.4)	52 (100.0)		0.002
No	443 (92.5)	69 (82.1)	72 (98.6)	–		

MD, mean difference between fast/slow groups with 95% confidence interval; p, comparison of fast/slow groups (chi-square test for nominal variables and t-test for continuous variables), ECD, extracapsular dissection.

Then two entities were compared in **Table 4**: recurrent tumors (r-PA) and those successfully treated. Patients with recurrence demonstrated significantly faster tumor growth in the last few years (44% in patients with recurrence vs. 20% in patients without recurrence, p < 0.001). There was no significant difference in age and tumor volume between recurrence groups.

**Table 4:** Comparison of features in r-PA versus PA.

Characteristic	Recurrence (Yes)	Recurrence (No)	MD (95% CI)	p	RFS, %	95% CI	Log-rank p
Total group, N	52	584			96.3	94.6–98.1	
Age, years, mean ± SD	48.04 ± 14.14	47.92 ± 15.01	0.12 (-4.36; 4.13)	0.958			
Sex, n (%)							
Female	29 (55.8)	383 (65.6)		0.205	97.5	95.8–99.2	0.100
Male	23 (44.2)	201 (34.4)			94.3	90.7–98.1	
Tumor volume [cm^3^], median (Q1;Q3)	2.00 (1.24;4.13)	2.34 (1.20;6.43)	-0.34 (-0.37; 0.90)	0.497			
Recent accelerated tumor growth, n (%)							
Yes	17 (43.6)	108 (19.9)		<0.001	92.6	87.1–98.6	0.020
No	22 (56.4)	438 (80.1)			98.0	96.6–99.5	
Margins, n (%)							
Negative	14 (29.2)	361 (71.6)		<0.001	98.5	97.0–100	<0.001
Positive	34 (70.8)	143 (28.4)			89.8	84.5–95.4	
capsular rupture*	0 (0.0)	36 (25.2)		<0.001	n/a	n/a	<0.001
tumor spillage*	1 (0.7)	38 (26.6)			n/a	n/a	
incomplete or bare capsule*	16 (11.2)	14 (9.8)			64.1	47.1–87.4	
absence of encapsulation in the pathology specimen*	7 (4.9)	37 (25.9)			93.1	84.3–100	
satellite nodules*	10 (7.0)	18 (12.6)			89.2	79.4–100	
FN status, n (%)							
Other	25 (48.1)	31 (5.3)		<0.001	96.2	94.3–98.1	0.700
Intact	27 (51.9)	553 (94.7)			98.0	94.3–100	
Tumor location by ESGS, n (%)							
I	31 (59.6)	273 (46.7)		0.102	96.7	94.3–99.2	0.700
Other	21 (40.4)	311 (53.3)			96.0	93.4–98.6	
II	35 (67.3)	336 (57.5)		0.221	95.6	93.1–98.3	0.500
Other	17 (32.7)	248 (42.5)			97.2	94.9–99.5	
III	16 (30.8)	74 (12.7)		0.001	91.4	84.4–99.0	0.020
Other	36 (69.2)	510 (87.3)			97.0	95.2–98.7	
IV	7 (13.5)	38 (6.5)		0.111	92.6	83.2–100	0.300
Other	45 (86.5)	546 (93.5)			96.6	94.8–98.3	
V	3 (5.8)	5 (0.9)		0.017	80.0	51.6–100	0.020
Other	49 (94.2)	579 (99.1)			96.5	94.8–98.2	
I-II	49 (94.2)	534 (91.4)		0.663	96.4	94.6–98.3	0.500
Other	3 (5.8)	50 (8.6)			95.0	88.3–100	
III-IV	17 (32.7)	96 (16.4)		0.006	93.5	88.0–99.3	0.100
Other	35 (67.3)	488 (83.6)			96.8	95.0–98.7	
I-IV	51 (98.1)	552 (94.5)		0.434	96.1	94.3–98.0	n/a
Other	1 (1.9)	32 (5.5)			n/a	n/a	
Parotidectomy type, n (%)							
Partial superficial	14 (26.9)	181 (31.0)		0.009	96.2	93.3–99.3	0.600
Superficial	11 (21.2)	153 (26.2)			98.1	95.6–100	
Total	12 (23.1)	42 (7.2)			92.6	83.2–100	
ECD	14 (26.9)	202 (34.6)			96.0	93.0–99.0	
ND	1 (1.9)	6 (1.0)			n/a	n/a	

MD, mean/median difference between groups with and without recurrence with 95% confidence interval; RFS, Kaplan Meier relapse-free survival; p, comparison of groups (chi-square test for nominal variables, t-test for age and Mann-Whitney U test for tumor volume); ND, no data available; *% frequency calculated to positive margins.

Thus, second, a multivariate analysis was performed. Using recurrence (yes/no) as the outcome variable, fast/slow categories as the predictor variable, and age, gender, margin, FN status as the covariates, multifactor logistic regression analysis showed that fast PAs significantly predicted recurrence vs. slow PAs (p = 0.035). Fast PAs were increasing the risk of recurrence 10-fold vs. slow PAs, exp β = 10.20, CI_95_ [1.66; 197.87]. Model assessment using Hosmer–Lemeshow GOF test (p = 0.743) confirmed good fit of the model to the data. Interpretation of logistic regression data for fast/slow categories indicates that in patients with fast PA, the risk of recurrence increases by 10.2-fold compared to patients with slow PA.

## Discussion

PA progression rate, differences in tumor growth rate, and impact on recurrence still remain unclear. In this study, the authors aimed to show that one of the clinical parameters—tumor growth rate—significantly correlates with a higher risk of recurrence. Despite the progress in this field, the exact causes of PA recurrence remain elusive. It has been hypothesized that the various reasons for PA recurrence can be grouped into pathology-related (capsule thickness or lack of capsule (21, 22), pseudopodia, satellite nodules (23, 24), and multi-centricity) and surgery-related factors such as rupture of the tumor, spillage of tumor contents, insufficient margins of resection due to nerve branches, and inadequate excision related to the type of surgery (25). Conceptually, re-growth of the tumor as a result of inadequate initial resection could be defined as PA persistence rather than PA recurrence. Owing to the time frame between the initial surgery and recurrence, it is generally implied that the re-operation is performed by a different surgeon who tends to blame the first inadequate procedure (25). In our setting, we can abandon the hypothesis that tumor re-growth results from inadequate surgery, as the 1,154 benign salivary gland tumors observed over a 10-year period were operated on by only two experienced surgeons.

The initial medical interview allowed us to derive data concerning the speed of tumor progression, and it is on this basis that the patient was advised on the pressing necessity to undergo surgery. Thus, the surgeon was able to make short- or long-term considerations and plan the procedure precisely according to these indications. Clinical observation has led us to distinguish a small group of PAs demonstrating clinical behavior that differs from the vast majority of PAs.

Fast PAs are characterized by an unexpectedly short medical history and relatively rapid growth. Additionally, they exhibit imaging features that, while similar to other PAs, are extremely exaggerated, that is, presenting jagged fragments instead a smooth tumor capsule, with only polycyclic pseudopodia and satellites. In a diametrically different group, we distinguished from typical PAs a group of tumors demonstrating even calmer biology, with very slow, long-term progression. Thus, we divided the whole PA group into three subsets of “fast,” “normal,” and “slow” tumors. The criteria for such division were based on several clinical and radiological features that differed in this seemingly homogenous benign PA group (25–29).

So far, two clinical features—patient age and tumor size—have been associated with a higher risk of recurrence, and this finding is coherent with most conclusions in the literature. Larger PAs have a tendency to exhibit incomplete capsules and are additionally associated with more numerous satellite nodules (9, 24).

Based on fast/normal/slow PA categorization, we proved that this clinical aspect is of great practical importance. Not only does it allow for preliminary selection of patients for immediate surgery, they are under greater vigilance during surgery and are more frequently monitored for relapse. Surgical access can be potentially modified, such as forgoing extracapsular access in rapid tumors in favor of parotidectomy. One may also consider a lower threshold for postoperative RT in the event of tumor spillage. We conduct follow-up visits once a year for all PAs, while select tumors demonstrating adverse findings are followed up every six months for a period of 10 years. It is of note that tumor development over a shorter period is also very probable (1, 27, 30).

Our publication delineating the clinical aspect of the course and speed of PA development is innovative and unique. It measurably defines the clinical distinctiveness of PAs. Every experienced surgeon is aware of this problem and probably intuitively schedules earlier surgeries and closely monitors rapid tumors. Nevertheless, we have proven that this feature is statistically more significant than other features for the development of recurrence, and on this basis we recommend careful and longer monitoring of these patients.

The main limitations of our study include inconsistent imaging examinations in our patients. Magnetic resonance imaging (MRI), ultrasonography (US), and computed tomography (CT) are the most commonly ordered studies for PA because these protocols describe the precise location and size of the tumor (31). However, some of the patients received US or MRI while some received CT. Another limitation of this study is patient-reported symptom duration, where we can broadly assume that symptom duration was underestimated by a few months.

## Conclusion

The simple clinical aspect of slow or fast PA development is of great practical importance and can constitute a surrogate of the final histopathological result derived from the surgical specimen. The slow or fast nature of the PA to some extent indicates prognostic features such as recurrence risk. This finding requires correlation with histological and molecular features in further stages of research.

## Data Availability Statement

The original contributions presented in the study are included in the article/supplementary materials. Further inquiries can be directed to the corresponding author.

## Ethics Statement

The studies involving human participants were reviewed and approved by Bioethics Committee of Poznan University of Medical Sciences (Resolution No. 781/16). The patients/participants provided their written informed consent to participate in this study.

## Author Contributions

Conceptualization, KP, MW. Data curation, KP, PK. Formal analysis, PK, JC. Investigation, KP, EB, JC. Methodology, KP, EB, MW. Project administration, MW. Resources, KP, PK, MW. Software, EB, JC. Supervision, MW. Validation, KP, EB, MW. Visualization, KP, JC. Writing—original draft, KP, JC, MW. Writing—review and editing, KP, JC, MW. All authors contributed to the article and approved the submitted version.

## Conflict of Interest

The authors declare that the research was conducted in the absence of any commercial or financial relationships that could be construed as a potential conflict of interest.
